# Rendezvous technique using a drill dilator through endosonographically created route in a patient with huge pancreatic stones

**DOI:** 10.1055/a-2641-1876

**Published:** 2025-07-25

**Authors:** Hirofumi Yamazaki, Yasunobu Yamashita, Yuki Kawaji, Takashi Tamura, Masahiro Itonaga, Reiko Ashida, Masayuki Kitano

**Affiliations:** 1 13145 Second Department of Internal Medicine, Wakayama Medical University, Wakayama, Japan

Endoscopic pancreatic stenting (EPS) is used for symptomatic chronic pancreatitis. Endoscopic ultrasound-guided pancreatic duct drainage (EUS-PDD) can create a route to the pancreatic duct instead of using EPS 1 , but stent migration frequently occurs 2 . In a novel rendezvous technique, a drill dilator was inserted through an endosonographically created route (ESCR) to help pass a stricture after EUS-PDD.

A patient with chronic pancreatitis had abdominal pain and huge pancreatic stones in the main pancreatic duct (MPD) ( Fig. 1 ). EPS via the major papilla failed due to MPD stricture caused by the stones, but EUS-PDD was performed to create an ESCR with a plastic stent ( Fig. 2 ).

**Fig. 1:**
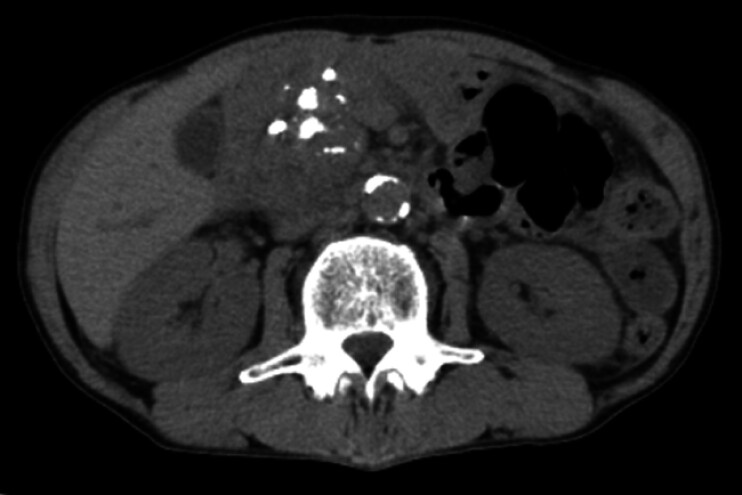
Computed tomography image showing huge pancreatic stones in the main pancreatic duct.

**Fig. 2:**
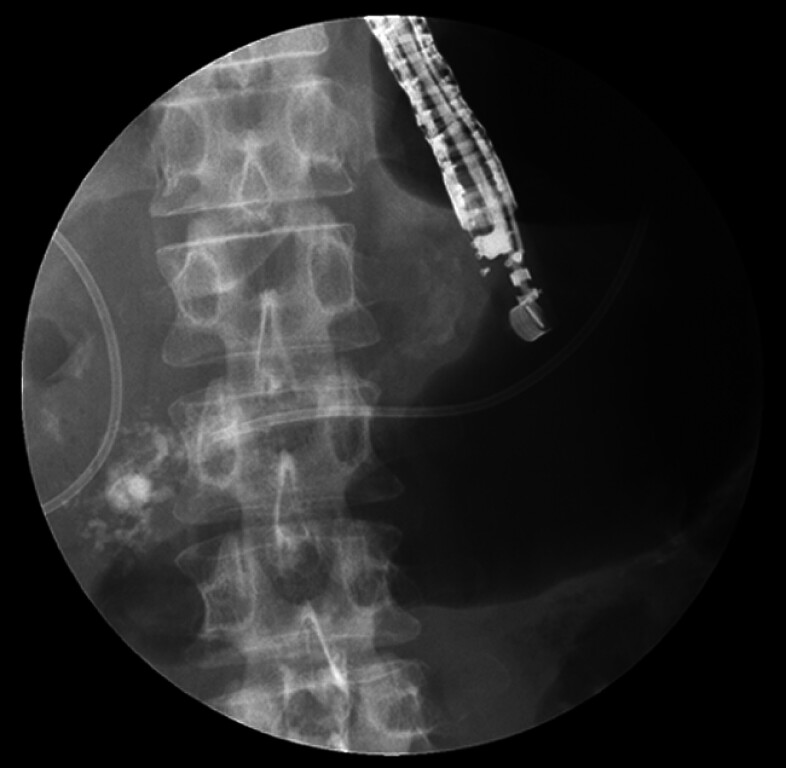
Placement of a plastic stent during initial endoscopic ultrasound-guided pancreatic duct drainage.

Later, we attempted a rendezvous technique with a drill dilator to insert a pancreatic duct stent through the papilla. The plastic stent in the EUS-PDD route was removed and a self-expandable metal stent was inserted into the ESCR ( Fig. 3 ). We planned to insert a catheter and guidewire through the ESCR, but the guidewire could not pass through stricture at the MPD. After electrohydraulic lithotripsy by pancreatoscope, the guidewire could pass, but not the catheter. We therefore inserted the drill dilator to dilate the MPD stricture, and the apex of the drill dilator reached the duodenal lumen through the minor papilla. The apex was kept in the duodenal lumen after scope removal. After inserting the scope to the descending part, the guidewire was inserted into the drill dilator lumen. Then, we simultaneously retrieved the drill dilator from the mouth while advancing a catheter over the guidewire, keeping it close to the apex of the drill dilater. Finally, a plastic stent could be inserted from the minor papilla ( Video 1 ).

**Fig. 3:**
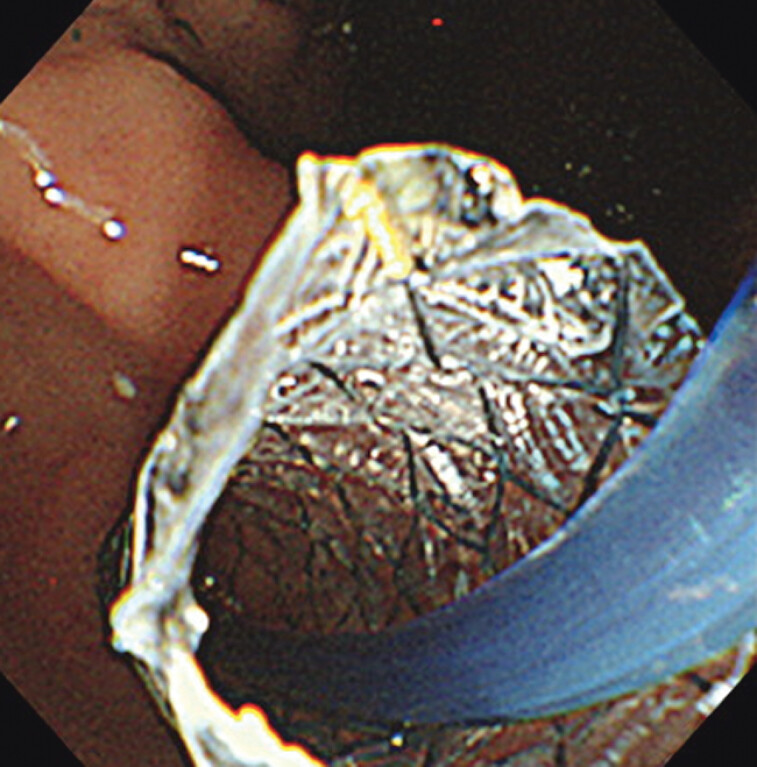
Insertion of a self-expandable metal stent via the endosonographically created route.

Rendezvous technique using a drill dilator through an endosonographically created route.Video 1

When EPS is difficult, a drill dilator can help to pass severe strictures via the rendezvous technique.

Endoscopy_UCTN_Code_TTT_1AS_2AD

## Notes

Endoscopy E-Videos https://eref.thieme.de/e-videos
*E-Videos* is an open access online section of the journal *Endoscopy* , reporting on interesting cases and new techniques in gastroenterological endoscopy. All papers include a high-quality video and are published with a Creative Commons CC-BY license. Endoscopy E-Videos qualify for HINARI discounts and waivers and eligibility is automatically checked during the submission process. We grant 100% waivers to articles whose corresponding authors are based in Group A countries and 50% waivers to those who are based in Group B countries as classified by Research4Life (see: https://www.research4life.org/access/eligibility/ ). This section has its own submission website at https://mc.manuscriptcentral.com/e-videos .
